# Trends in age‐related disease burden and healthcare utilization

**DOI:** 10.1111/acel.12861

**Published:** 2018-11-29

**Authors:** Vincenzo Atella, Andrea Piano Mortari, Joanna Kopinska, Federico Belotti, Francesco Lapi, Claudio Cricelli, Luigi Fontana

**Affiliations:** ^1^ Department of Economics and Finance University of Rome “Tor Vergata” Rome Italy; ^2^ Center for Health Policy Stanford University Stanford California; ^3^ CEIS Tor Vergata University of Rome “Tor Vergata” Rome Italy; ^4^ Italian College of General Practitioners (SIMG) Florence Italy; ^5^ Charles Perkins Center and Central Clinical School University of Sidney Sidney New South Wales; ^6^ Department of Clinical and Experimental Sciences Brescia University Brescia Italy

**Keywords:** aging, cancer, cardiovascular disease, chronic disease, diabetes, disease burden, healthcare expenditure, hypertension

## Abstract

Aging is a strong risk factor for many chronic diseases. However, the impact of an aging population on the prevalence of chronic diseases and related healthcare costs are not known. We used a prevalence‐based approach that combines accurate clinical and drug prescription data from Health Search CSD‐LPD. This is a longitudinal observational data set containing computer‐based patient records collected by Italian general practitioners (GP) and up‐to‐date healthcare expenditures data from the SiSSI Project. The analysis is based on data collected by 900 GP on an unbalanced sample of more than 1 million patients aged 35+, observed in different time periods between 2005 and 2014. In 2014, 86% of the Italian adults older than 65 had at least one chronic condition, and 56.7% had two or more. Prevalence of multiple chronic diseases and healthcare utilization increased among older and younger adults between 2004 and 2014. Indeed, in the last 10 years, average number of prescriptions increased by approximately 26%, while laboratory and diagnostic tests by 27%. The average number of DDD prescribed increased with age in all the observed years (from 114 in 2005 to 119.9 in 2014 for the 35–50 age group and from 774.9 to 1,178.1 for the 81+ patients). The alarming rising trends in the prevalence of chronic disease and associated healthcare costs in Italy, as well as in many other developed countries, call for an urgent implementation of interventions that prevent or slow the accumulation of metabolic and molecular damage associated with multiple chronic disease.

## 1 INTRODUCTION

Many of the developed and developing countries are facing an unprecedented and rapid rise in the number of elderly people that has far‐reaching health and economic implications (Fontana, Kennedy, Longo, Seals & Melov, 2014). Life expectancy has almost doubled in the last 150 years, increasing from ~45 years in 1,850 to ~80 years in many industrialized countries today (Christensen, Doblhammer, Rau & Vaupel, 2009). About 10% of the population was 65 or older in the mid‐1950s, with only 1%–3% being older than 80 (Christensen et al., 2009; Statistisches Bundesamt). Today, in countries like Italy, the proportion of people aged 65 and older has more than doubled (21.5%), and it is predicted to reach a worrisome 33% in 2050, while the proportion of the 85+ has more than tripled (5.9%), and it is predicted to reach 14% in the mid‐2050s ISTAT Geodemo (2017).

Aging is a well‐established risk factor for the development of multiple chronic diseases, including cardiovascular disease, stroke, cancer, osteoarthritis, and dementia (Christensen et al., 2009; Fontana et al., 2014). The diagnosis and treatment of these common chronic diseases place a significant burden on National Healthcare System budgets. As clearly stated in a report by the World Economic Forum and the Harvard School of Public Health (Bloom et al., 2011), experts are increasingly expressing concern that non‐communicable diseases (NCDs) will first put at risk the financial sustainability of all public healthcare systems, and then will result in long‐term macroeconomic impacts on labor supply, capital accumulation, and GDP worldwide. Data from the United States show that men and women with chronic conditions worked 6.1% and 3.9% fewer hours, respectively (Suhrcke, Nugent, Stuckler & Rocco, 2006), while a “healthy” lifestyle could reduce healthcare costs by 49% in the working age adults aged 40 and above (Mayer‐Foulkes, 2011). More important, NCDs also compromise future economic and human development because poverty and ill health are often passed down from one generation to the next.

Despite the relevance of the problem, little is known about the true effect of aging on the prevalence and trends of age‐associated chronic diseases and related healthcare costs using population data. Compared to the existing literature, this study proposes an important improvement by using a patient‐based approach with accurate clinical data collected from 2005 to 2014 by 900 Italian GP serving about 1.5 million patients per year, homogeneously distributed across Italian regions. To our knowledge, this is one of the largest longitudinal population sample ever used in this type of studies, based on detailed and objectively reported clinical outcomes. Moreover, our findings are obtained in a very favorable setting, since the Italian National Health System (NHS) provides universal and substantially free healthcare access to all citizens, with 87% of medical services publicly financed (OECD, 2013), avoiding problems of patient selection associated with insurance based healthcare systems.

## 2 METHODS

### 2.1 Sources of data

The clinical data have been extracted from the Health Search/CSD Longitudinal Patient Database, an Italian general practice registry, which collects data obtained from computer‐based patient records of a selected group of 900 general practitioners (GP), homogeneously distributed across all Italian regions. The work was carried out with no specific funding sources. The GPs voluntarily agree to collect patients’ information and to attend training courses for data entry (Lawrenson, Williams & Farmer, 1999). The Health Search database contains patient demographic data that are linked through an encrypted patient code with their medical records (diagnoses, prescribed tests, and tests results), drug prescription information (medication name, date of filled prescription, and number of days’ supply), self‐reported hospital admissions, and date of death. To be considered for participation in epidemiological studies, GPs meet “up to standard” quality criteria pertaining to the levels of coding, prevalence of well‐known diseases, mortality rates, and years of recording (Filippi et al., 2005). The Health Search database complies with European Union guidelines on the use of medical data for research and has been previously demonstrated to be a valid data source for scientific research (Atella et al., 2015). GPs collect the information on daily basis. However, for this study the records have been collapsed to obtain yearly information. The following ICD9 codes were included in the present study: Hypertension (401–405, 437.2), Atrial Fibrillation (427.3), Stroke (433–436, 437.0, 437.1, 438, 342), Heart Failure (428, 402.91, 404.91, 402.01, 402.11, 404.01), Acute Heart Attack (410), Acute Other Ischemia (411), Previous Heart Attack (412), Angina (413), Chronic Other Ischemia (414), Vascular Disease (440–442, 443.9, 445), Dyslipidemia (272.0, 272.1, 272.2), Diabetes (250, excluding 250.x1 and 250.x3), Cancer (150, 151, 153, 154, 155, 156, 157, 162, 174, 179, 182, 183, 185, 188, 189, 196, 197, 200, 202–208, 231.1, 231.2, 232.3–232.9, 233), COPD (491.2, 492), Parkinson (332), and Arthritis (714.0, 714.1, 714.3, 716.5–716.9).

The following Anatomical Therapeutic Chemical (ATC) Classification System codes have been included in this analysis: A02, A02A, A02BA, A02BC, A02D, A03BB, A03, A03F, A04, A05, A06, A07, A10 A10AB, A10AC, A10AD, A10AE, A10B, A10BA, A10BB, A10BD, B01, B03, B03XA01, B03XA02, B05, B05A, C01, C01A, C01B, C01D, C01E, C02, C02A, C02C, C02D, C03, C03A, C03B, C03C, C03D, C03E, C04, C07, C08, C09, C10, G03, G03H, G03X, G04, H01, H01AC01, H01CB01, H01CB02, H02, H02AB01, H02AB04, H02AB07, H03, H03A, H05, L01, L01AA, L01AD, L01B, L01C, L01X, L02, L03, L04, M01, M02, M04, M05, N02A, N02AA01, N02AA05, N02AA55, N02AA59, N02AA99, N02AX02, N02AX52, N02B, N02BA01, N04, N05, N05A, N05AA01, N05AA03, N05AD01, N05AF05, N05AH02, N05AH03, N05AH04, N05AH06, N05AL05, N05AL07, N05AN01, N05AX08, N05AX12, N05B, N05BA01, N05BA06, N05BA08, N05BA12, N05BA49, N05C, N05CD01, N05CD05, N05CD06, N05CD08, N05CF02, N06, N06A, N06AA09, N06AB03, N06AB04, N06AB05, N06AB06, N06AB10, N06AX05, N06AX11, N06AX16, N06AX18, N06AX21, N06D, N06DA02, N06DA03, N06DA04, N06DX0, R03, R03AC12, R03AC18, R03AK04, R03AK06, R03AK07, R03BA02, R03BA05, R03BB04, R03CC02, and R03DA04.

### 2.2 Statistical analysis

This study used a patient‐based approach combining healthcare utilization and accurate clinical data collected by GP. The sample consists of all the patients registered in the GP roster between 2005 and 2014. The same GPs are followed over time together with their patients but the latter can enter or exit the data set, due to change of residency, death or withdrawal, at any point in time. To guarantee the quality of the data and the reliability of the results, we have selected from the full list of patients only the active patients. We define the active population as the individuals aged between 15 and 95, who are not dead, who have not revoked their GP, and who have at least 2 years of observations. The analysis was conducted separately by four age classes (35–50‐year‐olds, 51–65‐year‐olds, 66–80‐year‐olds, and over 81‐year‐olds) and period of observation (2005–2014). First of all, to analyze the distribution of diseases by age class, the statistical analysis examined age‐specific prevalence rates of major NCDs and adverse health conditions responsible for a vast proportion of individual healthcare utilization, costs, and eventual mortality. Second, to understand the role that multiple diseases can have on a single patient, a comorbidity index was constructed as an un‐weighted sum of different conditions affecting each patient. The index was then analyzed conditionally on age class, gender, and year. Third, to study how the diseases affected the utilization of resources, we have age standardized the drug prescription patterns. We then computed average number of drugs prescribed by ATC code, comorbidity index, and year. Finally, for a better understanding of the origin of health expenditure, we analyzed the healthcare utilization by age class and comorbidity index and by type of healthcare delivered (drugs, GP visits, diagnostic tests, and specialist visits).

## 3 RESULTS

A total of 1,035,984 (485,876 males and 550,108 females) and 1,089,777 patients (524,183 males and 565,594 females) were included in the Health Search/CSD waves in 2005 and 2014, respectively (Table 1). The analysis was based on an unbalanced sample of patients aged 35+, observed at different times between 2005 and 2014. In 2005, the average age of the study sample was 48.9 years, and the gender composition was in favor of women in all age groups (53.1% in total and 67.1% for the 81+ group). Over a 10‐year period, the population sample has aged from 48.9 to 50.4 years, reflecting a 3.1% increase in age. The gender representation slightly changed from 53.1% of females in 2005 to 51.9% in 2014.

**Table 1:** Changes in prevalence rate by age class

	2005					2014					2005–2014 growth rate				
	35–50	51–65	66–80	Over 80	tot	35–50	51–65	66–80	Over 80	tot	35–50	51–65	66–80	Over 80	tot
Age	42.3	57.8	72.4	85.2	48.9	42.8	57.6	72.4	85.8	50.4	1.3%	−0.3%	0.0%	0.6%	3.1%
Female	51.5%	52.0%	57.0%	67.1%	53.1%	50.8%	51.1%	53.3%	64.9%	51.9%	−1.4%	−1.7%	−6.5%	−3.3%	−2.3%
Hypertension	9.5%	34.7%	56.7%	59.7%	23.7%	11.6%	39.2%	67.3%	77.5%	30.0%	22.1%	13.0%	18.7%	29.8%	26.6%
Atrial fibrillation	0.2%	1.2%	5.0%	9.4%	1.7%	0.4%	1.7%	7.3%	16.2%	2.9%	100.0%	41.7%	46.0%	72.3%	70.6%
Stroke	0.3%	1.9%	8.0%	14.0%	2.7%	0.5%	3.3%	13.2%	23.6%	4.9%	66.7%	73.7%	65.0%	68.6%	81.5%
Heart failure	0.1%	0.5%	2.3%	6.9%	0.9%	0.1%	0.6%	2.8%	9.7%	1.3%	0.0%	20.0%	21.7%	40.6%	44.4%
Heart attack (acute)	0.1%	0.9%	1.6%	1.9%	0.6%	0.2%	1.2%	2.4%	3.2%	1.0%	100.0%	33.3%	50.0%	68.4%	66.7%
Heart attack (old)	0.1%	0.8%	1.8%	1.9%	0.6%	0.1%	0.5%	1.5%	2.0%	0.5%	0.0%	−37.5%	−16.7%	5.3%	−16.7%
Angina	0.1%	0.7%	1.6%	1.9%	0.6%	0.2%	0.8%	2.1%	2.9%	0.8%	100.0%	14.3%	31.3%	52.6%	33.3%
Other ischemia (acute)	0.0%	0.1%	0.2%	0.2%	0.1%	0.0%	0.2%	0.5%	0.6%	0.2%	—	100.0%	150.0%	200.0%	100.0%
Other Ischemia (chronic)	0.4%	2.9%	7.5%	10.3%	2.6%	0.4%	3.2%	8.7%	12.5%	3.3%	0.0%	10.3%	16.0%	21.4%	26.9%
Vascular disease	0.3%	1.4%	4.3%	5.1%	1.4%	0.5%	2.5%	8.7%	11.5%	3.1%	66.7%	78.6%	102.3%	125.5%	121.4%
Dyslipidemia	4.0%	12.0%	15.6%	8.5%	7.2%	7.4%	21.3%	31.5%	24.2%	14.6%	85.0%	77.5%	101.9%	184.7%	102.8%
Diabetes	1.8%	9.0%	16.3%	15.2%	6.2%	2.1%	9.2%	21.0%	21.9%	8.0%	16.7%	2.2%	28.8%	44.1%	29.0%
Cancer	1.3%	4.5%	9.4%	10.6%	3.7%	1.6%	4.9%	11.8%	14.9%	4.8%	23.1%	8.9%	25.5%	40.6%	29.7%
COPD	0.3%	1.4%	4.2%	6.0%	1.5%	0.3%	1.1%	3.4%	5.5%	1.3%	0.0%	−21.4%	−19.0%	−8.3%	−13.3%
Rheumatoid arthritis	0.3%	0.7%	1.1%	1.0%	0.5%	0.4%	1.1%	1.6%	1.7%	0.8%	33.3%	57.1%	45.5%	70.0%	60.0%
Other arthritis	1.1%	1.8%	2.2%	2.1%	1.4%	1.9%	3.8%	5.3%	5.6%	3.0%	72.7%	111.1%	140.9%	166.7%	114.3%
Parkinson	0.0%	0.2%	1.1%	2.5%	0.4%	0.0%	0.2%	1.3%	3.3%	0.5%	—	0.0%	18.2%	32.0%	25.0%
Prescriptions	4.4	10.8	20.6	23.1	9.1	4.3	11.7	27.1	33.9	11.5	−2.3%	8.4%	31.7%	47.1%	26.4%
DDD prescribed	114.0	357.3	707.5	774.9	289.4	119.9	410.2	990.1	1,178.1	394.1	5.1%	14.8%	39.9%	52.0%	36.2%
Visits	0.6	1.1	1.7	0.9	0.9	0.4	0.7	1.3	1.2	0.7	−30.4%	−32.1%	−19.7%	28.6%	−22.6%
Lab tests and diagnostic	7.0	11.2	16.0	9.0	9.1	7.6	12.5	21.8	20.9	11.6	8.1%	11.5%	36.3%	131.3%	27.0%
Observations	295,806	230,004	178,927	55,886	1,035,984	329,025	260,336	189,817	73,807	1,089,777	11.2%	13.2%	6.1%	32.1%	5.2%

Hypertension and Dyslipidemia were the most common health conditions. Hypertension prevalence strongly increased with age, from 9.5% in the 35–50 age group to 59.7% in the 80+ age group. Similarly, prevalence of diabetes increased eightfold from 1.8% in the 35–50 age group to 15.2% in 80+ age group. This age gradient was similar for cancer and much stronger for COPD and all the cardiovascular diseases. In particular, for the youngest patients, the prevalence of atrial fibrillation, stroke, heart failure, heart attack, angina, and Parkinson's disease was negligible (<1.0%), reaching pronounced levels only for patients 66 and older.

As expected, healthcare services utilization was higher in the elderly than in younger age groups. The average number of prescriptions was more than eight times higher in the 81+ age group compared with the youngest group. The average prescription duration was more than three times longer for the oldest group, while doctor visits and prescription of laboratory and diagnostic tests were almost three times more frequent for the oldest group.

### 3.1 Trends in chronic diseases

**Figure 1:**
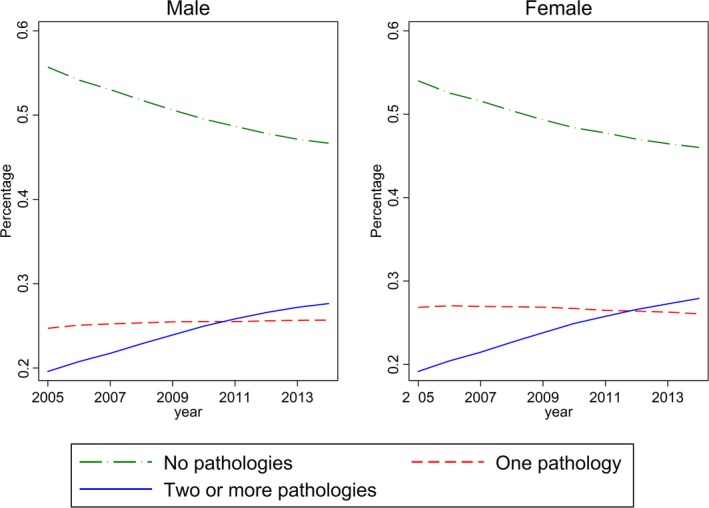
Percentage of patients by gender, number of chronic diseases, and wave

All the chronic diseases examined showed an increasing trend throughout the study period (Table 1). In particular, the prevalence of chronic diseases such as dyslipidemia, vascular disease, other ischemia (acute), and other arthritis more than doubled for the entire population sample (Table 1). The proportion of patients reporting no pathologies decreased from 25.8% in 2005 to 23.5% in 2014, whereas the proportion reporting only 1 chronic disease remained relatively stable (Figure 1). The proportion reporting 2 or more comorbidity rose sharply from 20.2% in 2005 to 28% in 2014.

**Figure 2:**
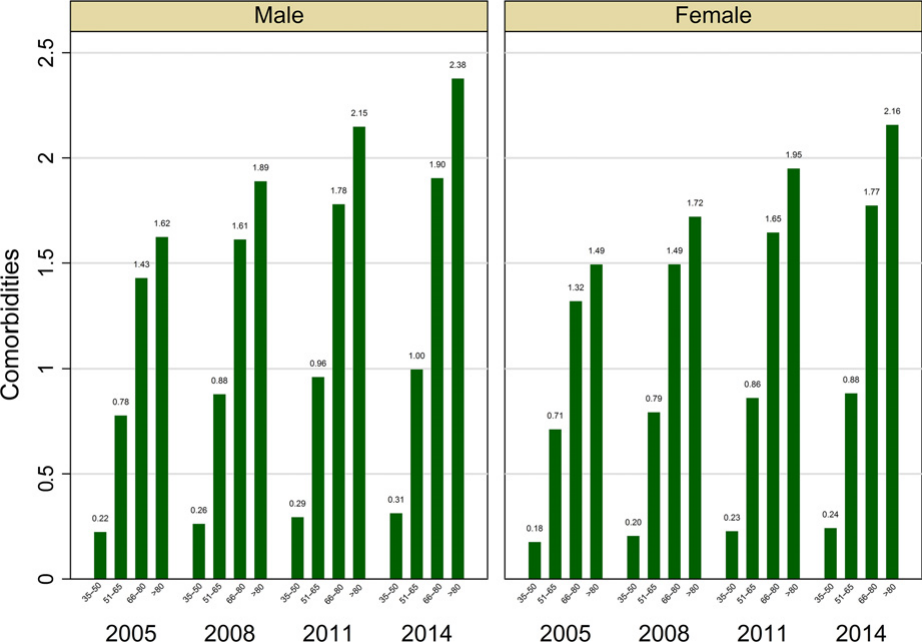
Average number of comorbidities by gender, age group, and wave

Figure 2 provides information on the average number of chronic diseases by age group and gender. The average number of comorbidities increased monotonically with age in all the observed years, i.e. from 0.22 in 2005 to 0.31 in 2014 for the 35–50 age group and from 1.62 to 2.38 for the 80+ males. Table 2 shows the evolution of the share of patients with different number of chronic conditions by age and gender. Large differences in comorbidity levels exist among age groups. More than 75% of males aged 35–50 had no pathologies in 2005. This proportion dropped with age to 20% for the 80+ group. Between 2005 and 2014, the prevalence of “no comorbidities” patients (having none or only one disease) decreased for all age groups in both male and female patients. While the decrease was the sharpest, with almost 60% drop for patients aged 80+, it was relatively important also in the youngest patients (−7%). Interestingly, we observed a sharp increase in the prevalence of patients with four or more comorbidities for all age groups, with both the 66–80 and 80+ groups exhibiting more than a twofold increase.

**Table 2:** Percentage of patients by number of chronic diseases, age, gender, and wave

	Age group	2005			2008			2011			2014		
		All (%)	Female (%)	Male (%)	All (%)	Female (%)	Male (%)	All (%)	Female (%)	Male (%)	All (%)	Female (%)	Male (%)
No pathologies	35–50	78.70	80.39	76.90	76.17	78.21	74.03	74.20	76.61	71.64	72.97	75.71	70.12
	51–65	49.83	50.05	49.58	45.79	46.41	45.13	42.76	43.63	41.82	41.47	42.75	40.14
	66–80	27.07	26.78	27.44	22.52	22.14	22.97	19.01	18.87	19.17	16.03	15.84	16.24
	>80	20.38	20.15	20.75	15.16	15.03	15.38	11.46	11.43	11.50	8.41	8.41	8.42
One	35–50	17.53	16.66	18.44	19.12	18.03	20.27	20.44	19.21	21.74	21.38	19.80	23.02
	51–65	32.42	33.52	31.23	33.33	34.27	32.31	33.62	34.45	32.74	33.91	34.61	33.18
	66–80	35.46	37.36	33.08	33.93	35.70	31.81	31.92	33.56	30.04	30.70	32.18	29.11
	>80	33.09	35.25	29.46	30.40	32.43	27.14	26.96	28.78	24.19	24.38	26.12	21.89
Two	35–50	3.12	2.55	3.72	3.82	3.19	4.48	4.36	3.48	5.29	4.59	3.73	5.48
	51–65	12.69	12.45	12.96	14.40	14.12	14.70	15.91	15.65	16.18	16.43	15.96	16.91
	66–80	22.74	23.28	22.06	25.01	26.07	23.74	26.70	27.86	25.35	27.76	29.18	26.23
	>80	25.28	25.72	24.54	27.02	27.87	25.66	28.04	29.40	25.97	28.37	29.93	26.14
Three	35–50	0.53	0.34	0.72	0.71	0.51	0.92	0.80	0.60	1.02	0.84	0.64	1.05
	51–65	3.73	3.19	4.32	4.62	4.01	5.28	5.40	4.72	6.12	5.69	5.00	6.41
	66–80	9.83	8.94	10.95	11.84	11.11	12.71	13.70	13.18	14.30	15.20	14.82	15.62
	>80	13.13	12.29	14.53	16.06	15.34	17.22	18.50	17.95	19.34	20.44	20.32	20.62
Four or more	35–50	0.13	0.06	0.21	0.18	0.07	0.30	0.20	0.10	0.31	0.22	0.12	0.32
	51–65	1.33	0.80	1.91	1.86	1.19	2.58	2.32	1.55	3.13	2.50	1.68	3.35
	66–80	4.90	3.65	6.47	6.70	4.98	8.75	8.67	6.52	11.14	10.30	7.98	12.80
	>80	8.13	6.58	10.73	11.35	9.32	14.61	15.04	12.43	19.00	18.39	15.21	22.94

### 3.2 Trends in healthcare utilization

Over the 10‐year study period, we observed a substantial increase in outpatient healthcare utilization (drug prescriptions, laboratory and diagnostic tests, and doctor visits). The average number of prescriptions increased by 26.4% and laboratory tests and diagnostic by 27% (Table 1).

**Figure 3:**
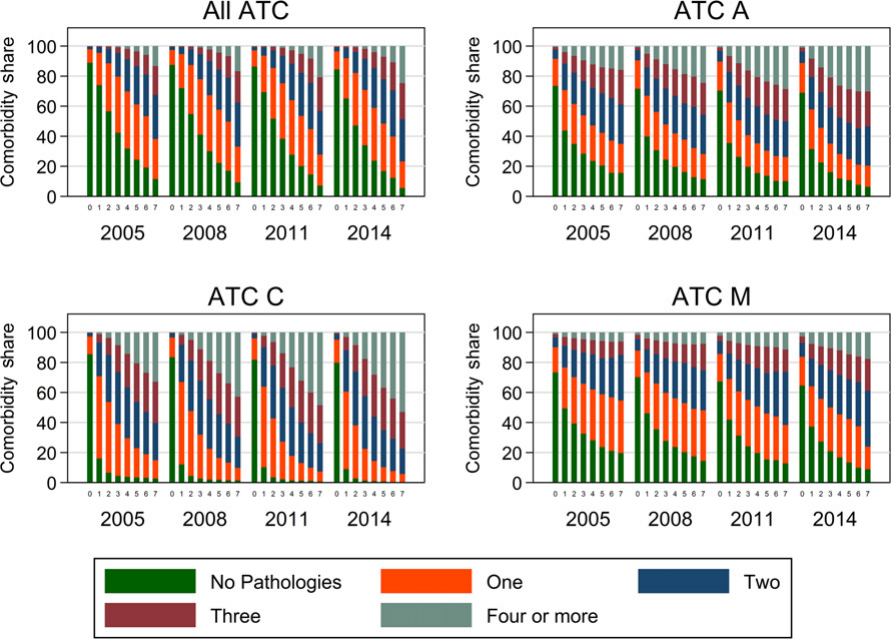
Share of comorbidities by number of drugs prescribed to patients, ATC class, and wave

Figure 3 shows a strong association between the number of comorbidities and the number of drugs prescribed. This association increased across waves, and it was heterogeneous across different ATC classes. Ranging from 0 to 7+ drugs prescribed belonging to all ATC types, the prevalence of patients with no disease or no comorbidities decreased drastically, while the proportion of patients with 3, 4, or more comorbidities increased exponentially. This pattern was particularly pronounced for cardiovascular medications (ATC C), while the drug‐comorbidity index nexus was relatively weaker for musculoskeletal drugs (ATC M).

## 4 DISCUSSION

Our findings demonstrate that the prevalence of multiple chronic diseases and healthcare utilization increased over time among older adults in Italy. In 2014, 86 percent of Italian adults older than 65 years now live with at least one chronic condition and 56.7 percent have more than one. Consistently, our data show a substantial increase in outpatient healthcare utilization, i.e. drug utilization, diagnostic tests, and doctor visits, in both males and females. In the last 10 years, average number of prescriptions increased by approximately 26%, while laboratory and diagnostic tests by 27%. They account for a relevant fraction of the public healthcare budget every year (about 23 billions of euros and 20% of the total budget). Without concerted strategic preventive interventions, these numbers are expected to increase and become unsustainable, because the Italian total population over the age of 65 is projected to increase sharply over the next 15–20 years reaching a worrisome 33% (ISTAT Geodemo).

Such problems, however, are not limited to Italy. According to the Institute for Health Metrics and Evaluation, in 2013 NCDs (i.e. cardiovascular diseases, cancer, diabetes mellitus, chronic respiratory diseases, and musculoskeletal disorders) were responsible for the vast majority of deaths and healthcare costs in Europe as well (Institute for Health Metrics and Evaluation, 2013). Among these, cardiovascular diseases are the leading cause of death and are responsible for about half of all deaths in Europe, and cancer is the second main cause of mortality and healthcare costs. Similar data apply to USA and other advanced economies (Bleich et al., 2015; WHO, 2005). Consistently, in our study we found that the prevalence of hypertension, dyslipidemia, and diabetes, which are major risk factors for cardiovascular disease, have increased in the last few years together with the prevalence of heart failure, coronary heart disease, heart attack, stroke, vascular disease, and atrial fibrillation. We found a strong age‐dependent relationship between the prevalence of these diseases and age, which is problematic in light of the forecasted increase in the number of elderly people in Italy and many other industrialized countries.

Our results are in agreement with other studies, showing that more than 90% of older adults are affected by at least one chronic disease, and 60% have two or more chronic diseases (Hung, Ross, Boockvar & Liu, 2011). This increasing number of older adults with several comorbidities, which are at greater risk for disability, consume multiple drugs and spend longer time in hospitals, make health care more complex and expensive by increasing the need for organized, multidisciplinary care both within and outside hospitals (Vogeli et al., 2007; Wolff, Starfield & Anderson, 2002). Consistently, in our study we found that drug consumption and healthcare costs increased dramatically with age. Moreover, we found a strong relationship between the number of comorbidities and the number of drug prescribed to patients across all the different ATC classes. This pattern was particularly pronounced for cardiovascular medication costs that in 2014 accounted for 4,087 million of euros (3,631 million of which were sustained by the Italian National Health Service) (Osservatorio sull'impiego dei medicinali).

In many European countries as well as in US more than 60% of healthcare spending is caused by people affected by several chronic diseases (Wolff et al., 2002). Our findings suggest that the problem of multiple chronic conditions is not restricted to older adults, but is rising also in men and women younger than 65 years old, probably because of the growing epidemic of overweight and obesity‐associated chronic diseases due to excessive calorie intake, unhealthy diets, and lack of physical activity (Atella et al., 2015; Fontana & Hu, 2014; Heymsfield & Wadden, 2017). Thus, patients are exposed at an earlier onset of chronic diseases, which combined with longer life expectancy, extends the time period of living in bad health (Mulye et al., 2009; Olshansky et al., 2005). While Italy's adult obesity problem might be considered mild in comparison with other countries, childhood excess body weight rates are notoriously considered one of the highest (36% for boys and 34% for girls) among all the OECD countries (Ng et al., 2014). This is a major problem for Italy and many other developed and developing countries worldwide because individuals with obesity are more likely to develop a wide range of chronic diseases, which have a huge economic toll on both direct and indirect healthcare costs (Atella et al., 2015; Fontana & Hu, 2014; Heymsfield & Wadden, 2017).

It is important to highlight the strengths and limitations of this study. The use of objective clinical data (e.g. true health expenditure, chronic disease diagnosis, test results, drug prescriptions, outpatient diagnostic tests, specialist visits, and hospital admissions) collected for a large sample of patients and entered in an up‐to‐date computer‐based database by trained GPs is a major strength of this study. In addition, it is important to stress that the Italian NHS is a public and universalistic system, which provides substantially free health services for all citizens. According to OECD Health Data, in 2012 about 87% of medical services in Italy was publically financed. This setting favors the external validity of the study and minimizes the selection problems related to the presence of private insurance plans. It is important to highlight that the results of this study most likely reflect real changes in population health status rather than changes in physician's diagnostic and prescribing behaviors. Indeed, several other studies have reported a worsening of health status in recent years (Atella et al., 2017; Case & Deaton, 2015; Hu, Yuan, Rao, Zheng & Hu, 2014; Hulsegge et al., 2013; HwaJung & Schoeni, 2017). One major limitation of this study, however, is the lack of socioeconomic status indicators, which could potentially benefit the analysis and provide additional insights.

## 5 CONCLUSIONS

In conclusion, the findings of this study show that multiple chronic diseases have become increasingly prevalent not only among older but also in younger adults. Approximately 90% of Italian older adults have at least one chronic disease and more than 60% have more than one. Moreover, a growing number of individuals aged <65 are affected by one or more chronic conditions. Our data show that caring for all these people is expensive and unsustainable on the long‐term, because patients with multiple chronic conditions are at greater risk for disability, activity limitations, poor functional status, hospitalization, adverse drug events and are higher users of medical care services than those with 1 or no chronic conditions. The development of a range of lifestyle (e.g. healthier diets, physical activity, and tobacco and alcohol control activities) and pharmacological interventions that prevent the accumulation of metabolic and molecular damage with age are warranted to stop, or at least, delay this unsustainable demographic crisis of increased chronic diseases, disability, and escalating healthcare costs.

## CONFLICT OF INTEREST

The authors declare that they have no competing interests.
